# Supplementary material for: The Significance of FilmArray Blood Culture Identification Panel (FA-BCID) for Managing Patients with Positive Blood Cultures
Source: Diagnostics (Basel). 2023 Oct 29;13(21):3335. doi: 10.3390/diagnostics13213335 (PMC10648013; doi:10.3390/diagnostics13213335)
Supplement: Supplementary file 1 [file diagnostics-13-03335-s001.zip › diagnostics-2663948-supplementary.pdf]

Table S1. Microorganisms identified by MALDI-TOF and FA-BCID.

Sample no.	Gram Stain	FA BCID Result		MALDI-TOF Result		Concordance	Antibiotic resistance genes
		Identification	Mono/polymicrobial	Identification	Mono/polymicrobial		
**1**	G(+) cocci in chain	*Enterococcus Pseudomonas aeruginosa*	poly	*Enterococcus faecalis Pseudomonas aeruginosa*	poly	Y	
**2**	G(+) cocci in chain	*Streptococcus*	mono	*Streptococcus anginosus*	mono	Y	
**3**	G(+) cocci in chain G(-) bacilli	*Enterococcus*	mono	*Enterococcus faecium Shewanella algae*	poly	N	
**4**	Yeast	*Candida tropicalis*	mono	*Candida tropicalis*	mono	Y	
**5**	G(+) cocci in chain	*Enterococcus*	mono	*Enterococcus faecalis*	mono	Y	
**6**	G(-) rod	*Escherichia coli*	mono	*Escherichia coli*	mono	Y	
**7**	G(+) cocci in chain	*Enterococcus*	mono	*Enterococcus faecium*	mono	Y	
**8**	G(+) cocci in chain	*Streptococcus*	mono	*Streptococcus constellatus*	mono	Y	
**9**	Yeast	*Candida tropicalis*	mono	*Candida tropicalis*	mono	Y	
**10**	G(+) cocci in pairs Yeast	*Enterococcus Candida tropicalis*	poly	*Enterococcus faecium Candida tropicalis*	poly	Y	
**11**	G(+) cocci in chain G(-) rod	*Streptococcus*	mono	*Streptococcus intermidius*	mono	Y	
**12**	G(-) rod	*Escherichia coli*	mono	*Escherichia coli*	mono	Y	
**13**	G(-) rod	*Escherichia coli*	mono	*Escherichia coli*	mono	Y	
**14**	G(-) rod	*Klebsiella pneumoniae*	mono	*Klebsiella pneumoniae*	mono	Y	
**15**	G(-) rod	*Klebsiella pneumoniae*	mono	*Klebsiella pneumoniae*	mono	Y	
**16**	G(-) rod	*Escherichia coli Klebsiella pneumoniae*	poly	*Escherichia coli Klebsiella pneumoniae*	poly	Y	
**17**	G(+) cocci in cluster	*Staphylococcus aureus*	mono	*Staphylococcus aureus*	mono	Y	
**18**	G(-) rod	*Escherichia coli*	mono	*Escherichia coli*	mono	Y	
**19**	G(-) rod	*Escherichia coli*	mono	*Escherichia coli*	mono	Y	
**20**	G(-) rod	*Escherichia coli*	mono	*Escherichia coli*	mono	Y	
**21**	G(-) rod	*Escherichia coli*	mono	*Escherichia coli*	mono	Y	
**22**	G(-) rod	*Escherichia coli Klebsiella oxytoca*	poly	*Escherichia coli Klebsiella oxytoca*	poly	Y	
**23**	G(-) rod	*Escherichia coli*	mono	*Escherichia coli*	mono	Y	
**24**	G(-) rod	*Staphylococcus aureus* (mecA+)	mono	*Staphylococcus aureus*	mono	Y	mecA+

**25**	G(-) rod	*Escherichia coli*	mono	*Escherichia coli*	mono	Y	
**26**	G(+) cocci in cluster	*Staphylococcus aureus*	mono	*Staphylococcus aureus*	mono	Y	
**27**	G(-) rod	*Escherichia coli*	mono	*Escherichia coli*	mono	Y	
**28**	G(+) cocci in cluster	*Staphylococcus aureus* (mecA+)	mono	*Staphylococcus aureus*	mono	Y	mecA+
**29**	G(-) rod	*Escherichia coli* (KPC+)	mono	*Escherichia coli*	mono	N	KPC+
**30**	G(+) cocci in cluster	*Staphylococcus aureus* (mecA+)	mono	*Staphylococcus aureus* *Staphylococcus epidermidis*	poly	Y	mecA+
**31**	G(-) rod	*Klebsiella pneumoniae*	mono	*Klebsiella pneumoniae*	mono	Y	
**32**	G(-) rod	*Klebsiella pneumoniae*	mono	*Klebsiella pneumoniae*	mono	Y	
**33**	G(+) cocci in chain G(+) rod	None	-	*Enterococcus faecalis* *Clostridium tertium*	poly	N	
**34**	G(+) cocci in pairs	*Staphylococcus aureus* (mecA+) *Proteus*	poly	*Staphylococcus epidermidis*	mono	N	mecA+
**35**	G(+) cocci in cluster	*Staphylococcus aureus*		*Staphylococcus aureus*	mono	Y	
**36**	G(-) rod	*Escherichia coli* *Proteus*	poly	*Escherichia coli*	mono	N	
**37**	G(-) rod	*Enterococcus* *Escherichia coli* *Pseudomonas aeruginosa*	poly	*Escherichia coli*	mono	N	
**38**	G(-) rod	*Escherichia coli* *Proteus*	poly	*Escherichia coli*	mono	N	
**39**	G(+) cocci in pairs	*Streptococcus pneumoniae* *Proteus*	poly	*Streptococcus pneumoniae*	mono	N	
**40**	G(-) rod	*Escherichia coli* *Proteus*	poly	*Escherichia coli*	mono	N	
**41**	G(-) rod	*Klebsiella pneumoniae*	mono	*Klebsiella pneumoniae*	mono	Y	
**42**	G(-) rod	*Escherichia coli*	mono	*Escherichia coli*	mono	Y	
**43**	G(-) rod	*Escherichia coli*	mono	*Escherichia coli*	mono	Y	
**44**	G(-) rod	*Escherichia coli*	mono	*Escherichia coli*	mono	Y	
**45**	G(-) rod	None	-	*Bacteroides fragilis*	mono	N	
**46**	G(-) rod	*Klebsiella pneumoniae*	mono	*Klebsiella pneumoniae*	mono	Y	
**47**	G(-) rod	*Escherichia coli*	mono	*Escherichia coli*	mono	Y	
**48**	G(+) cocci, G(-) rod	*Enterococcus* *Staphylococcus aureus* (mecA+) *Streptococcus agalactiae* *Escherichia coli*	poly	*Streptococcus agalactiae*	mono	N	mecA+
**49**	G(-) rod	*Escherichia coli*	mono	*Escherichia coli*	mono	Y	
**50**	G(-) rod	*Klebsiella pneumoniae* (KPC+)	mono	*Klebsiella pneumoniae*	mono	N	KPC+

**51**	G(+) cocci in cluster	*Staphylococcus aureus* (mecA+)	mono	*Staphylococcus caprae*	mono	N	mecA+
**52**	G(-) rod	*Escherichia coli* *Proteus*	poly	*Escherichia coli*	mono	N	
**53**	G(-) rod	*Proteus*	mono	*Citrobacter youngae*	mono	N	
**54**	G(-) rod	*Escherichia coli*	mono	*Escherichia coli*	mono	Y	
**55**	G(-) rod	*Klebsiella oxytoca*	mono	*Klebsiella oxytoca*	mono	Y	
**56**	G(+) cocci in cluster	*Staphylococcus* (mecA+)	mono	*Staphylococcus epidermidis*	mono	Y	mecA+
**57**	G(+) cocci in chain	*Streptococcus agalactiae* *Proteus*	poly	*Streptococcus agalactiae*	mono	N	
**58**	G(-) rod	*Escherichia coli*	mono	*Escherichia coli*	mono	Y	
**59**	G(+) cocci in pairs, G(-) rod	*Enterococcus* *Enterobacteriaceae*	poly	*Enterococcus faecium* *Citrobacter freundii*	poly	Y	
**60**	G(-) rod	*Escherichia coli*	mono	*Escherichia coli*	mono	Y	

Abbreviation: FA-BCID; filmArray Blood culture identification, MALDI-TOF; matrix-assisted laser desorption/ionization time-of-flight; KPC, Klebsiella penumoniae carbapenemas; mecA; methicillin resistance gene.
